# Commentary: The Predictive Processing Paradigm Has Roots in Kant

**DOI:** 10.3389/fnsys.2017.00098

**Published:** 2018-01-08

**Authors:** Majid D. Beni

**Affiliations:** Management, Science, and Technology, Amirkabir University of Technology, Tehran, Iran

**Keywords:** predictive processing, Kant, ecological psychology, inferentialism, Bayesianism

In a recent contribution, Swanson (2016) mentioned several reasons for understanding the predictive processing theory (PP) as a new development of Kant's theories of cognition. The aim of this general commentary is not to deny that PP has roots in Kant, but to stress the point that such an apparently innocent historical observation is laden with significant philosophical presuppositions. Swanson's interpretation neglects some significant philosophical discussions of PP. And being negligent of the divergence of philosophical views on PP may result in a distorted or misleading understanding of PP. This commentary aims to amend.

It is certainly possible to construe PP along the lines of Kantianism. The most important resemblances consist of emphasis on the “top-down” generation of percepts, the role of “hyperpriors,” and an inferential account of the brain's capacity to track causal structure in the world using only sensory data. Swanson also highlights the historical course of the transfer of Kantian ideas to PP through works of Hermann van Helmholtz. PP stemmed out of Helmholtz's statistical theories of perception as unconscious inference. According to this reading, PP is centered on the idea of inferential links between the statistically constructed models and their external world targets. It models the brain as an inferential engine which uses Bayesian mechanisms to reduce the discrepancy between environment and its (i.e., the brain's) models of the environment. Accordingly, hierarchical top-down processing, generative models and the free energy principle could be construed in terms of inferentialism. To show that this construal could be related to Kant's theories, Swanson took great pains to delve into Kant studies and referred to works of Kant, Kitcher, Allison, and Brooks. Other philosophers have remarked that there is an affinity between PP and Kant's ideas too (Clark, 2013; Gładziejewski, 2016). Jakob Hohwy is the most prominent champion of the Kantian-cum-inferentialist cause. Hohwy's construal underscores the internalist nature of PP and highlights the inferentialist essence of the brain-world relationship. The brain generates *internal* models and uses predictive error reducing mechanisms to *infer* the evolving structure of the distal environment. It relies on Bayesian mechanisms to update its posterior beliefs and to infer the hidden causes of the sensory inputs from the *inside of the skull* (Hohwy, 2013, p. 220), and the brain is “secluded” and “skull-bound” (Hohwy, 2014, p. 1). This interpretation is in line with the spirit of Kantian modesty. The feature of the noumenal domain (i.e., the domain of things in themselves, i.e., things independent from their knowable phenomenal attributes) could be inferred only from inside the phenomenal domain. The important point is that, despite its virtues, this construal of PP is not the only game in the town, and there are other interpretations (which have been overlooked by Swanson). For example, Clark's (Clark, 2015, 2016a,b) construal has been developed along the lines of extended mind and embodied cognition theses. Swanson cited Clark in her paper, but unfortunately, the references to Clark are a bit too selective. Clark's construal presents PP along the lines of ecological psychology and pragmatism to underscore action-oriented nature of the brain's information processing and its dynamical interaction with the world. This alternative construal seems to be gaining momentum recently (see Bruineberg et al., 2016).

It is worth mentioning that this enactivist, ecological construal relies on the free-energy formulation of PP, which explains predictive processing in terms of the error reducing ability of the organisms that can move and sample their own sensations. Free energy is a thermodynamic quantity. It is defined by Friston as an information-theoretic measure that bounds or limits (by being greater than) the surprise on sampling some data, given a generative model (Friston, 2010, p. 1). It could be used to provide a biologically viable formulation of PP. Biological systems are exposed to random and unpredictable fluctuations in their environment. They can restrict themselves to occupying a limited number of states. Organisms engage in predicting the future outcomes and by doing so they simultaneously build the causal models of the structure of their local environment. This helps them to predict what will happen and be ready to encounter surprising violations of those predictions (Karl, 2012). It is possible to formulate active inferences in terms of the free-energy principle (Gallagher and Allen, 2016, p. 9). This explains how the embodied organism interacts with the environment to both generate probabilistic predictions that maximize survival (minimize free energy), and act on the world in a way that conforms sensory information to prior predictions (Gallagher and Allen, 2016). Therefore, the ecological interpretation draws on the role of active inferences and free energy principle to construe PP in light of works of John Dewey and James J. Gibson, rather than Kant (also see Bruineberg and Rietveld, 2014; Bruineberg et al., 2016). Needless to say that this ecological, environmental approach can be used for offering evolutionary and naturalistic arguments that are absent in the Kantian construal. The enactivist, ecological construal underscores the interconnection of cognitive mechanisms and sensorimotor and motor system. And it resolves issues that cannot be handled by the Kantian construal. For example, Swanson submitted that “PP's probabilistic and evolutionary approach (not to mention its computational and neuroscientific underpinnings) goes beyond Kant's insights” (2016, p.11). But the probabilistic and evolutionary aspects of PP could be easily related to the evolutionary and information-theoretic context of ecological psychology. So, Swanson's claim cannot be presented as a brute historical fact. There are philosophical presuppositions at issue, and Swanson's construal highlights some aspects and underplays other aspects (e.g., the role of action, the evolutionary basis, connection to Shannon information^1^, etc.). This is not just a pedantic observation. The consequences of choosing each of the alternative interpretations could be scientifically and philosophically significant. I shall elaborate.

Swanson's discussion reached its climax when he points out that Kant and PP each aim to offer detailed accounts of how minds track “hidden causes” using only the data from the senses, and they both develop these accounts using methods of top-down analysis in an attempt to reverse-engineer perception and cognition (Swanson, 2016, p. 4 ff.). While this construal could be true, it cannot accommodate a robust realist theory of the relation between the structure of stimuli and the causal structure of the world. As Hohwy conceded, the brains cannot crawl out of their shells (i.e., skulls) to garner independent evidence for the veracity of the inferences (Hohwy, 2014). Therefore, some form of skepticism about the truth of the brain's judgments concerning the features of the external world is bound to endure. Ecological psychology, on the other hand, dispenses with the mechanism of judgment and assumes that perceivable ambient information is sufficient for action-perception, and mechanisms of inference and judgment are superfluous. According to the ecological construal of PP, the perceptual system engages the world that is parsed according to our organism-specific needs and action repertoire rather than representing the mind-independent world from behind an inferential veil (i.e., the barrier between the brain's internal models and the environment) (Clark, 2016b, p. 195). This construal includes no inferences, hidden causes, or evidentiary boundaries^2^, which do not contribute to enabling the cognizer to realize the causes of sensation. Therefore there remains no room for skepticism. Moreover, an ecological construal of PP could deal with some vague aspects of the statistical inferences, in a way that remains beyond the scope of a Kantian construal. Below, I shall briefly instantiate this point.

Within PP account prediction error is reduced by perceptual inference. And in a Bayesian framework, we have to rely on prior information to form reliable opinion about the hidden causes of perceptions. But critics of Bayesianism have put their finger on the problem of the subjective nature of the prior probabilities of Bayesian conditionalization. However, the ecological construal could rely on the active inferences and free-energy principle to explain how the priors of Bayesian equations themselves are optimized (Friston, 2010, p. 3). According to Friston, minimizing the free energy effectively optimizes empirical priors. That is to say, “because empirical priors are linked hierarchically, they are informed by sensory data, enabling the brain to optimize its prior expectations online” (Friston, 2010). Given the hierarchical arrangement of cortical sensory areas (Felleman and Van Essen, 1991; Friston, 2010, p. 3), it is possible to assume that the biological brain itself uses these hierarchical models to optimize its priors through dynamical interaction with the environment. This accommodates biological realism about mechanisms of Bayesian conditionalization. Since optimization of the priors is a result of dynamical interactive mechanisms (which allow for an ecological construal), it can be argued that the priors of Bayesian conditionalization are not assigned subjectively. Similarly, Orlandi (2016) has argued that Bayesian theories *per se* do not constitute good solutions to the inverse problem^3^, and that they are not constructivist in nature. This means that the orthodox versions of predictive processing, which aim to account for how a statistical system (e.g., visual system) derives a single percept from underdetermined stimulation encounter a problem. But as Orlandi argued, reinforcing Bayesianism with the Gibsonian insight would help us address the inverse problem in a better way. Namely, the ecological emphasis on studying the statistical regularities of the environment could shed a new light on tackling the underdetermination problem. It should be added that despite Orlandi's radical departure from the goals of representationalism, the dichotomy between inferential and ecological approaches does not need to be underscored. Many an advocate of the ecological construal sought to traverse the gap between ecological and inferential approaches. For example, Clark who emphasized the role of embodiment of the organism on many occasions, denied that it is possible to account for error reduction in entirely non-representational terms, and without invoking the concept of a hierarchical probabilistic generative models (Clark, 2015, pp. 5–6, Clark, 2016b, p. 293). For other cases of this reconciliatory approach see (Allen and Friston, 2016; Dolega, 2017).

None of these is meant to question the value of Swanson's construal of PP. Nor do I suggest that the ecological construal is correct and Kantianism about PP is wrong (for, ecological psychology is haunted by demons of its own too). I just wanted to highlight the point that Swanson's theory is based on (at times questionable) philosophical presuppositions which exaggerate some aspects of PP and distort some others. We may still embrace the Kantian construal, but we need to know that it domesticates PP to certain philosophical presuppositions (as does its ecological alternative). And none of these interpretations represents PP without certain distortions.

## Author contributions

The author confirms being the sole contributor of this work and approved it for publication.

### Conflict of interest statement

The author declares that the research was conducted in the absence of any commercial or financial relationships that could be construed as a potential conflict of interest.

## Footnotes

^1^Shannon's notion of information, defined in terms of Mathematical Theory of Communication, offers to determine the amount of uncertainty or ‘disorder’ *H(X)* in a collection of messages in terms of a probability distribution *P* over the set of messages (Shannon and Weaver, 1949). According to this theory, the communication entropy of *X* for a set of messages *x*_*i*_ = (*I*= 1,… n) is:

(1) $$H\left(X\right)=-\sum_{i=1,n}P\left(x_{i}\right)\text{log }P\;(x_{i})$$

^2^This is the boundary between the evidence and hypothesis. It is evidentiary because it is defined by the occurrence of the evidence, boundary because causes beyond it can be only inferred (Hohwy, 2014, p. 264).

^3^Inverse problem is the problem of how to “infer back from the effects on the sensory system to the causes in the world” (Hohwy, 2013, p. 53–54). Given that one effect may be caused by a number of different causes, the problem could be formulated as a problem of underdetermination.
